# Hydrogel nanocomposite based on alginate/zeolite for burn wound healing: *In vitro* and *in vivo* study 

**DOI:** 10.22038/IJBMS.2023.68897.15016

**Published:** 2023

**Authors:** Hadi Samadian, Reza Vahidi, Majid Salehi, Hossein Hosseini-Nave, Arman Shahabi, Saeed Zanganeh, Mahla Lashkari, Seyedeh Mehrnaz Kouhbananinejad, Nariman Rezaei kolarijani, Seyed Mohammad Amini, Majid Asadi-shekari, Mohamad Javad Mirzaei Parsa

**Affiliations:** 1 Research Center for Molecular Medicine, Hamadan University of Medical Sciences, Hamadan, Iran; 2 Research Center for Hydatid Disease in Iran, Kerman University of Medical Sciences, Kerman, Iran; 3 Department of Tissue Engineering, School of Medicine, Shahroud University of Medical Sciences, Shahroud, Iran; 4 Sexual Health and Fertility Research center, Shahroud University of Medical Sciences, Shahroud, Iran; 5 Tissue Engineering and Stem Cells Research Center, Shahroud University of Medical Sciences, Shahroud, Iran; 6 Department of Microbiology and Virology, School of Medicine, Kerman University of Medical Sciences, Kerman, Iran; 7 Cell Therapy and Regenerative Medicine Comprehensive Center, Kerman University of Medical Sciences, Kerman, Iran; 8 Department of Hematology and Laboratory Sciences, Faculty of Allied Medical Sciences, Kerman University of Medical Sciences, Kerman, Iran; 9 Student Research Committee, School of Medicine, Shahroud University of Medical Sciences, Shahroud, Iran; 10 Radiation Biology Research Center, Iran University of Medical Sciences, Tehran, Iran; 11 Neuroscience Research Center, Institute of Neuropharmacology, Kerman University of Medical Sciences, Kerman, Iran; 12 Department of Medical Nanotechnology, Faculty of Allied Medical Sciences, Kerman University of Medical Sciences, Kerman, Iran

**Keywords:** Alginate, Clinoptilolite, Hydrogel, Nanocomposite, Wound healing, Zeolite

## Abstract

**Objective(s):**

The main objective of the current assay was to evaluate the antibacterial and regenerative effects of hydrogel nanocomposite containing pure natural zeolite (clinoptilolite) integrated with alginate (Alg) as wound healing/dressing biomaterials.

**Materials and Methods:**

The zeolites were size excluded, characterized by SEM, DLS, XRD, FTIR, and XRF, and then integrated into Alg hydrogel followed by calcium chloride crosslinking. The Alg and alginate zeolite (Alg/Zeo) hydrogel was characterized by swelling and weight loss tests, also the antibacterial, hemocompatibility, and cell viability tests were performed. In animal studies, the burn wound was induced on the back of rats and treated with the following groups: control, Alg hydrogel, and Alg/Zeo hydrogel.

**Results:**

The results showed that the hydrodynamic diameter of zeolites was 367 ± 0.2 nm. Zeolites did not show any significant antibacterial effect, however, the hydrogel nanocomposite containing zeolite had proper swelling as well as hemocompatibility and no cytotoxicity was observed. Following the creation of a third-degree burn wound on the back of rats, the results indicated that the Alg hydrogel and Alg/Zeo nanocomposite accelerated the wound healing process compared with the control group. Re-epithelialization, granulation tissue thickness, collagenization, inflammatory cell recruitment, and angiogenesis level were not significantly different between Alg and Alg/Zeo nanocomposite.

**Conclusion:**

These findings revealed that although the incorporation of zeolites did not induce a significant beneficial effect in comparison with Alg hydrogel, using zeolite capacity in hydrogel for loading the antibiotics or other effective compounds can be considered a promising wound dressing.

## Introduction

The human body’s skin has various vital functions and it is considered the largest organ of the body requiring critical care. Any damage to the skin should be treated with effective treatments. Accordingly, planetary studies have been conducted to develop treatment/healing biomaterials with the highest efficacy (1-3). Wound healing is a complicated and multistage process including hemostasis, inflammation, proliferation, and maturation. Various types of biomaterials have been proposed and developed as wound healing/dressing materials. Among them, hydrogels have shown promising results due to their fascinating properties. Hydrogels are hydrated 3D structures fabricated from hydrophilic polymers with the ability to absorb a huge amount of water without the dissolution of polymer chains (4-7). 

According to the wet wound-healing hypothesis, wound healing/dressing materials with the ability to provide moisture in the wound bed can provide better treatment outcomes than other structures. Moreover, hydrogels can absorb wound exudates due to the high water absorption capacity and subsequently improve the healing process. Hydrogels can be fabricated with different synthetic and natural polymers (3, 8). Among them, natural polysaccharides, such as chitosan, dextran, and alginate (Alg) have shown promising results due to good biocompatibility, acceptable biodegradation profile, and presence of cell adhesion sites in their structure. Alg is an anionic polysaccharide made from two copolymers, mannuronic acid, and guluronic acid, with promising properties such as high viscosity, high stability, and gelling properties (9, 10). Moreover, Alg can be physically cross-linked using divalent cations (e.g., Ca^+2^) forming a hydrogel structure (11, 12). It has been reported that the application of Alg as the wound healing/dressing material resulted in fascinating healing outcomes. Alg can stimulate/activate monocytes and macrophages to produce tumor necrosis factor α (TNF-α) and interleukin-6 (IL-6) and to accelerate chronic wound healing (13, 14).

Moreover, nanostructured materials strongly affect the healing process due to their effects on cell behavior, proliferation, migration, and differentiation. Accordingly, researchers have been evaluating nanocomposites as the scaffold in regeneration medicine and observed promising results. Various types of nanostructures and materials have been employed as fillers in the nanocomposite structures. Zeolites are multi-aspect, hydrated crystalline aluminosilicates with micro-and nano features and it has been reported that the application of zeolites can improve the wound healing process. The incorporation of zeolites into the biomaterials can accelerate oxygen delivery to cells and improve bioactivity and biocompatibility (15-17). Clinoptilolite, a natural zeolite, has various applications in agriculture, industries, medicine, and animal husbandry due to having unique properties such as high availability, low cost, and high surface area/volume ratio (18). It has been mentioned that modified zeolites have an antibacterial activity which is obtained by the ion-exchange process (19, 20). However, as natural zeolites are extracted mainly from volcanic and sedimentary rocks, they are different in composition and morphology from one deposit to another (21). Moreover, adverse reactions or reduction of expected activity of zeolites are associated with minerals in zeolites (22), also different Al/Si ratios in various zeolite types showed different antimicrobial effects against bacteria, yeast, and fungi (20). Some researchers evaluate the antibacterial effects of unmodified/pure zeolite due to different compositions that zeolites may have had. Therefore, in this study nanocomposite comprising alginate and zeolite (clinoptilolite) were fabricated to evaluate the role of unmodified zeolite-embedded hydrogel in antibacterial or wound healing experiments. 

## Materials and Methods

***Materials***

Micronized Iranian clinoptilolite (Anzymite®) powder was obtained from Afrand Tooska (Tehran, Iran). Carboxymethyl cellulose (CMC, low viscosity, MW), sodium alginate, and calcium chloride (CaCl_2_) were purchased from Sigma-Aldrich (St. Louis, MO, USA). The MTT assay kit (Roth, Germany), Fetal Bovine Serum (FBS, Gibco, Germany), DMEM/F-12 cell culture medium (Gibco, Germany), Trypsin-EDTA (Gibco, Germany), Penicillin-Streptomycin (Gibco, Germany), Ketamine and Xylazine (Alfasan, Netherlands) were used for the biological evaluations. The cells (L929 fibroblast cell line) and animals (Male adult Wistar rats) were obtained from the National Cell Bank of Iran (NCBI), Pasteur Institute of Iran) Tehran, Iran). 

***Zeolite purification/size exclusion***

The purification and size exclusion of the zeolites were carried out using sedimentation and centrifugation. Briefly, 20 g of zeolite was dispersed in 500 ml of deionized (DI) water and stirred for 2 hr at room temperature. Then, the mixture was centrifuged at 1000 rpm for 90 sec and the supernatant was filtered through the filter paper and the resulting solution was dried at 180 °C for 24 hr. The dried zeolite was used for characterization and hydrogel nanocomposite fabrication. 

***Zeolite characterization***

Scanning electron microscopy (SEM) was used to visualize the purified zeolites. The zeolite powder was coated with a thin layer of gold and imaged at the ambient conditions at an accelerating voltage of 20 kV. The hydrodynamic size of zeolites was performed via Dynamic Light Scattering (DLS, Cordouan Technologies). The crystallinity of zeolite and hydrogel zeolite was characterized using an X-ray diffractometer (XRD, PHILIPS PW1730, Netherlands) equipped with Cu Kα radiation (Kα = 1.54056 Å) and operating at 40 kV and 30 mA, step size 0.05◦ in scattering angle range from 2° to 70°. The XRD peaks were examined against data that have been provided before (23). The presence of specific chemical groups in the zeolite and hydrogel zeolite was determined by Fourier transform infrared spectroscopy (FTIR, Thermo AVATAR, USA). FTIR spectra were obtained in the range of 4000-400 cm^-1^ at a resolution of 4 cm^−1^. The X-ray fluorescence (XRF) technique was applied to quantitatively assess the elemental analysis of zeolite. 

***Hydrogel nanocomposite fabrication***

The purified zeolites were used to fabricate hydrogel nanocomposite as the dispersed phase and Alg as the matrix/continuous phase. A proper amount of the purified zeolites was added to DI water to obtain 2 % w/v and stirred for 24 hr at room temperature and sonicated several times in an ultrasonic bath to properly disperse the zeolites. Then, Alg was added to the mixture at the concentration of 3.5 % w/v and stirred for 12 hr to completely dissolve Alg. The cross-linking of Alg polymer chains was conducted using CaCl_2_ (70 mmol) and through physical crosslinking. The fabricated Alg/Zeo hydrogel nanocomposite was washed in DI water for 24 hr, frozen at -20 °C for 24 hr, and freeze-dried at -78 °C for 48 hr. The freeze-dried hydrogel nanocomposite was used for characterization. 

***Hydrogel nanocomposite characterization***

The internal structure and pore morphology of the Alg/Zeo hydrogel was evaluated using SEM. The freeze-dried hydrogel nanocomposite was crushed, sputter-coated with gold (a thin layer), and imaged under 20 kV accelerating voltage. 

The swelling kinetics of the hydrogels was assessed using the gravimetric method in DI water. The dry hydrogels were weighed (W0) submerged in DI water and incubated for 24 hr at 37 °C. At the specific time points, the wet hydrogels were removed and weighed (W1). The swelling kinetics was calculated using Equation 1 (Eq_1_). 

$\mathrm{Swelling}(\%)=(\frac{\mathrm{W1}-\mathrm{W0}}{\mathrm{W0}})\times 100$

The weight loss of the hydrogel was measured in phosphate-buffered saline (PBS, pH: 7.4) using Equation 2. The hydrogels were weighed (W0) submerged in PBS and incubated for 7 days at 37 °C. At the specific time points, the wet hydrogels were removed and weighed (W1). The weight loss was calculated using Equation 2 (Eq_2_). 

$\mathrm{Weight loss}(\%)=(\frac{\mathrm{W0}-\mathrm{W1}}{\mathrm{W0}})\times 100$

***Antibacterial studies***

The micro-broth dilution method, using Mueller Hinton broth, was used to evaluate the minimal inhibitory concentration (MIC) of the zeolite. Briefly, concentrations of 1 to 4 mg/ml of zeolite were prepared and poured into a 96-well plate, then, the bacteria with the concentration of 1.5 × 10^6^ colony-forming units/ml (CFU/ml) was added to each well. After incubation of the plate for 24 hours at 37 °C, the antimicrobial activity was defined against four reference strains including two Gram-positive bacteria [*Staphylococcus aureus (*ATCC 25923*) *and* Enterococcus faecalis *(ATCC 29212)] and two Gram-negative bacteria [*Escherichia coli *(ATCC 25922) and *Pseudomonas aeruginosa *(ATCC 27852)]. Positive control (culture medium containing the bacteria without zeolite) and negative control (culture medium without bacteria) were also prepared. Gentamicin was used as the control for anti-bacterial screening. The lowest concentration of zeolite which is completely capable of inhibiting bacterial growth was considered as MIC value. The MIC assays were carried out in triplicate against each bacterial strain to confirm the value of MIC for each tested bacteria.

***Hemocompatibility assay***

Incubation of the hydrogel nanocomposite and zeolite with red blood cells (RBCs) and measuring the induced hemolysis were conducted to investigate hemocompatibility. Blood was diluted with PBS (pH: 7.4) and 300 µl of the diluted blood was incubated with 5 mg of hydrogel nanocomposite or zeolite for 1 hr at 37 °C. After centrifugation of blood at 1500 rpm, the absorbance of the supernatant was read at 540 nm using a microplate reader (ELX808, Biotek, USA). Equation 3 (Eq_3_) was applied to calculate the hemolysis percent. 

$\mathrm{Hemolysis}(\%)=(\frac{\mathrm{Dt}-\mathrm{Dnc}}{\mathrm{Dpc}-\mathrm{Dnc}})\times 100$

D_t_: absorbance of blood incubated with hydrogel, D_nc_: absorbance of blood diluted with PBS, and D_pc_: absorbance of blood lysed with DI. 

***Cell viability investigation***

The indirect MTT assay was conducted to investigate the cytotoxicity of hydrogel nanocomposite towards the viability of L929 fibroblast cells. Briefly, different weights of zeolite and hydrogel nanocomposite were incubated in 1 cc DMEM/F-12 culture medium containing 10% FBS and 1% penicillin/streptomycin for 48 hr at 37 °C. Then, 5,000 cell/well was seeded in a 96-well tissue culture plate at 37 °C. After 24 hr the cell culture medium was replaced with the hydrogel nanocomposite extracts and incubated for 24 hr at 37 °C in 95% humidified air and 5% CO_2_. Finally, cell viability was measured using the MTT assay. 

***Wound induction***

For animal studies, 18 male Wistar rats with a weight of 220–250 g were purchased from Pasteur Institute (Tehran, Iran). The experiments were approved by the ethical committee of the Kerman University of Medical Sciences (IR.KMU.REC.1400.479) and conducted according to the university’s guidelines. The animals were randomly divided into three groups: control (wound without treatment), pure Alg hydrogel, and Alg/Zeo hydrogel, with 6 rats in each group. The animals were anesthetized by intraperitoneal injection of the sedating drug (Xylazine (6–8 mg/kg) and Ketamine (70–100 mg/kg) mixture). The hair of the back of the rats was shaved, sterilized, and a 2.5 cm diameter burn wound was induced using a hot (105 °C) bar (300 g) with a contact time of 8 sec. Then, the treatments were carried out according to the grouping. The macroscopic (for wound contraction evaluation) and microscopic (for histopathological evaluations) imaging were conducted on days 7 and 14 post-treatment. The wound closure percentage was calculated using Equation 4 (Eq_4_). 

$\mathrm{Wound closure}(\%)=(\frac{\mathrm{A0}-\mathrm{A1}}{\mathrm{A0}})\times 100$

Where A0 is the wound area on day zero and A1 is the wound area on day 7 or 14. 

***Healing process evaluation***

On postoperative days 7 and 14, rats were sacrificed, the complete wound was removed, and then fixed in 10% formalin solution. 5 μm sections were prepared for each sample and stained with hematoxylin and eosin (H&E). Finally, the epidermal and basal cell layers, granulation tissue thickness, and angiogenesis were reported in accordance with the criteria listed in the previous study (24). 

For further evaluation, the percentage of neutrophils, macrophages, and collagen deposition was determined. Indeed, the parameter-covered areas (%) per high power field were introduced as parameter percentages.

***Statistical analysis***

The obtained data were analyzed using SPSS 10.0 (IBM, NY, USA) by using a two-tailed paired Student’s t-test. To compare the means of quantitative variables between groups, Kruskal-Wallis and Dunnett tests were used. The data is reported as mean ± SD. A *P*-value<0.05 was considered statistically significant. 

**Figure 1:**
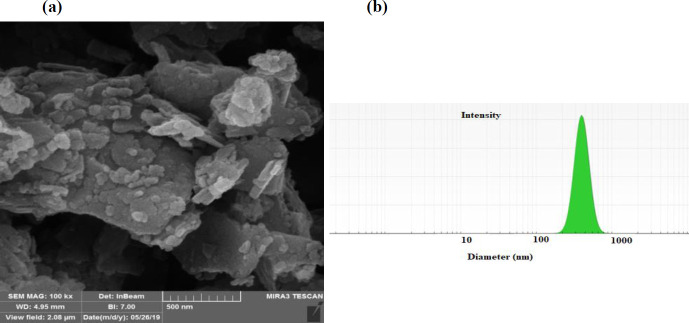
SEM micrograph of synthesized zeolites (a) and hydrodynamic light scattering diameter of zeolite (b)

**Figure 2:**
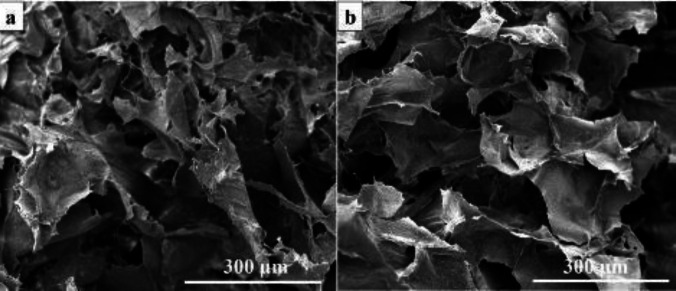
SEM micrograph of the fabricated hydrogel. (a) Pure Alg hydrogel and (b) Alg/Zeo hydrogel

**Figure 3:**
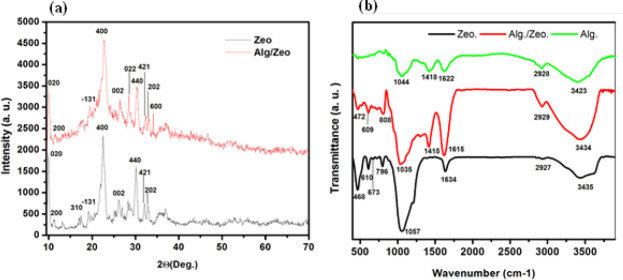
Structural characteristics of clinoptilolite X-ray diffraction (a). Fourier transform infrared spectra of Alginate, Zeolite, and Alg/Zeo hydrogel (b)

**Figure 4:**
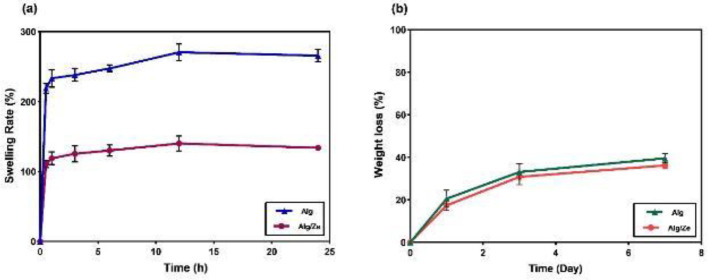
Swelling rate of the fabricated hydrogel nanocomposites (a), weight loss profile of the hydrogels in PBS solution (pH: 7.4) during 7 days (b)

**Figure 5:**
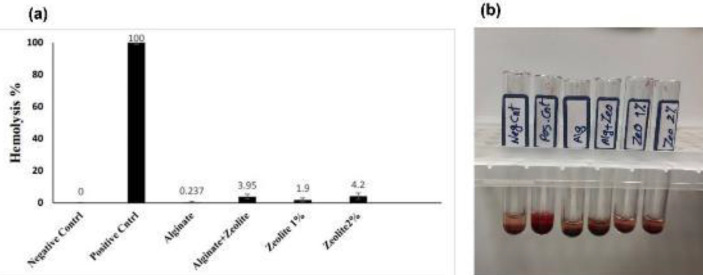
Percentage hemolysis (a) and optical photography (b) induced by the pure Alg, pure zeolite, and Alg/Zeo hydrogel

**Figure 6:**
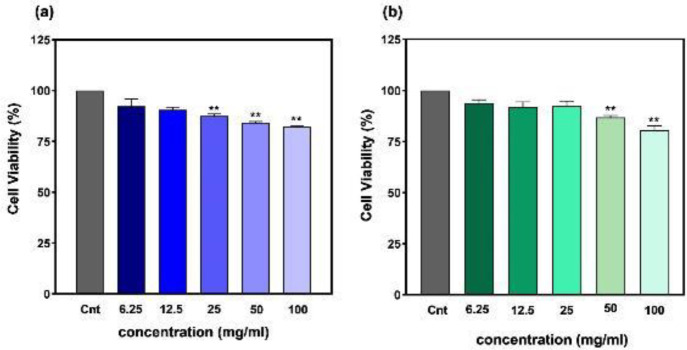
Viability of L929 fibroblast cells under incubation with (a) pure zeolite and (b) Alg/Zeo hydrogel nanocomposite. Measured by the indirect MTT assay. **P<0.01

**Figure 7:**
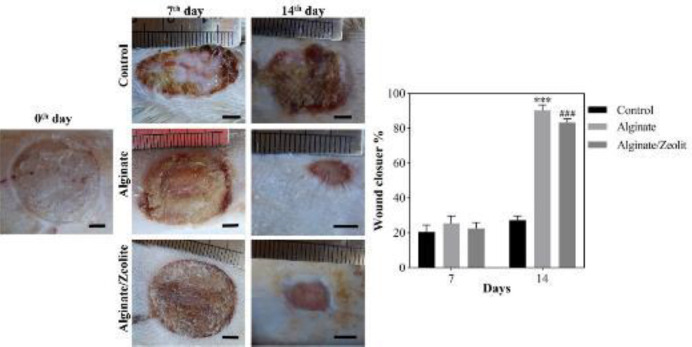
Macroscopic images and wound closure percentage of the burn wound under treatment with the hydrogels. Scale bars represent 5 mm. ***P<0.001

**Figure 8:**
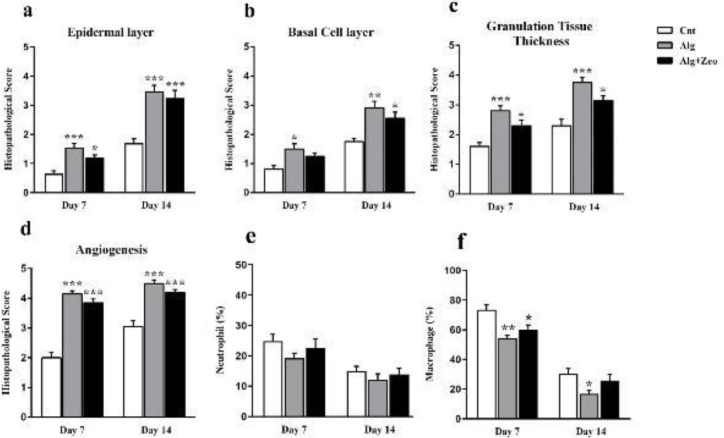
Quantitative histopathological analysis of the epidermal layer (a), basal cell layer (b), granulation tissue thickness (c), angiogenesis (d), and neutrophil and macrophage counts (e and f) in the studied groups. Wounds treated with Alg showed higher re-epithelialization, granulation tissue thickness, and angiogenesis levels as compared with other wounds. The macrophage percentage in the Alg group was significantly different from that in the Cnt group on the 7th (*P*<0.01) and 14th days (*P*<0.05). Compared with the Cnt group, Alg+Zeo-treated wounds exhibited a significant decrease in macrophage recruitment on the 7th day after injury (*P*<0.05). However, there was no significant difference in the number of these cells on the 14^th^ day (*P*>0.05). With respect to the neutrophil count, there was no significant difference in any groups during the study (*P*>0.05). Cnt: control group, Alg: Alginate group, and Alg+Zeo: Alginate/Zeolite group. Data are presented as mean±SEM. *P<0.05, **P<0.01, and ***P<0.001

**Figure 9:**
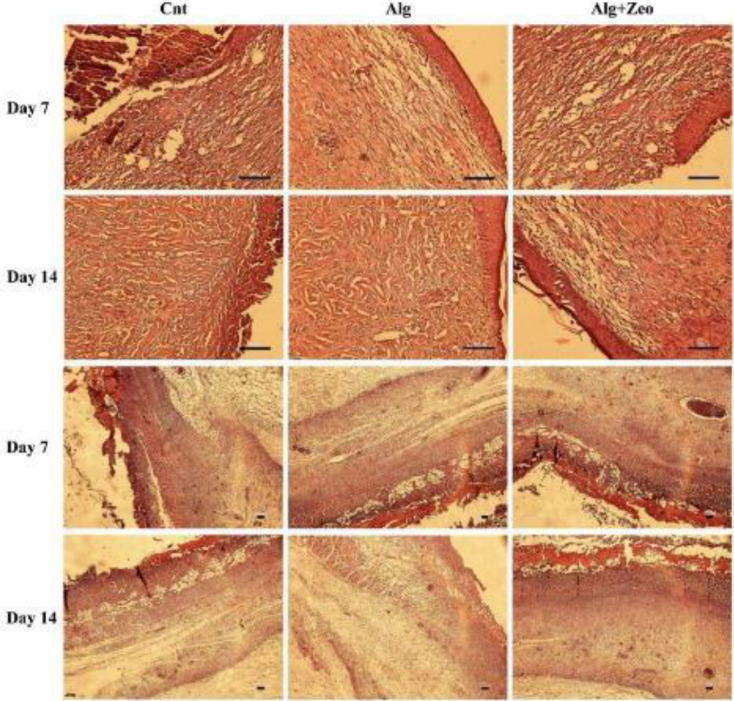
Representative H&E images of the wound healing process in different groups. Cnt: control group, Alg: Alginate group, and Alg+Zeo: Alginate/Zeolite group. Scale bar: 100 μm

**Figure 10:**
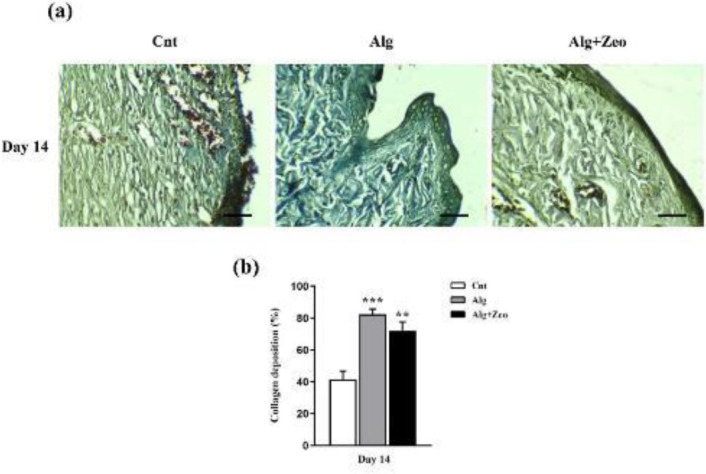
Assessment of wound collagenization rate (using Masson’s trichrome staining) after different treatments (a) and quantitative histopathological analysis of the collagenization (b). Scale bar: 100 μm. Cnt: control group, Alg: Alginate group, and Alg+Zeo: Alginate/Zeolite group. Data are presented as mean±SEM. **P<0.01 and ***P<0.001

## Results

***Zeolite characteristics***

The purified and size-excluded zeolites were characterized using SEM and DLS and the results are presented in Figure 1. The SEM image of the zeolites (Figure 1a) showed that the conducted size exclusion process separated the large-size zeolites and provided zeolites even at the nanoscale size. This feature is beneficial for fabricating homogenous nanocomposites. 

According to DLS (Figure 1b), the hydrodynamic size of the zeolites was 367 ± 0.2 nm. Baghbanian *et al*. prepared natural clinoptilolite nanoparticles mechanically by planetary ball mill and reported an average size of around 70 nm for the resulting structure (25). The XRF results showed that the obtained zeolite contains SiO_2_ (72.2%), Al_2_O_3_ (9.5%), Fe_2_O_3_ (2.6%), CaO (1.5%), Na_2_O (2.7%), MgO (1.8%), K_2_O (0.9%), TiO_2_ (0.8%), MnO (0.01%), and P_2_O_5_ (0.04). 

***Hydrogel nanocomposite properties***

The internal morphology and structure of the fabricated hydrogels were visualized using SEM imaging and the results are presented in Figure 2. The results illustrated that the fabricated hydrogels have a porous structure with interconnected pores architecture. 

The crystallinity of the fabricated hydrogel nanocomposite was also evaluated using the XRD analysis and the results are shown in Figure 3a. The results showed that the characteristic peaks of the zeolite evident in the fabricated hydrogel. In the clinoptilolite XRD pattern, the sharp peaks appeared at 2θ=9.85° and 2θ=22.4° corresponding to the Miller indexes of (020) and (004), respectively (23, 26). Based on the Sherrer-Deby equation the crystalline size of the synthesized clinoptilolite was estimated to be 41 nm. Moreover, there are not any peaks related to the Alg polymer which may be due to the amorphous nature of this polymer. On the other hand, the composite of zeolite-hydrogel had typical crystallite reflections due to the zeolite component. The presence of characteristic peaks of the zeolite that appeared in the hydrogel composites confirms the presence of zeolite in the nanocomposite structures. 

The FTIR spectrum of zeolite exhibits broadband at 3435 cm^−1^, which can be related to the stretching vibration of the hydroxyl groups in the Al-OH-Al and Si-OH-Si networks. The peak at 1634 cm^−1^ can be attributed to the H-O-H bending mode and the peak at 1058 cm^−1^ is related to Si-O-(Si), (Al) stretching vibration (27, 28). The characteristic bands of Alg were located at 1622 cm^−1^ and 1418 cm^−1^ which were assigned to the C=O stretching of the carboxyl group of sodium alginate. The peaks at 1044 cm^−1^ and 2928 cm^−1^ are assigned to –C–O–C stretching vibration of its polysaccharide structure and C-H stretching, respectively. The broadband at 3423 cm^-1^ is due to the stretching absorption of the hydroxyl groups of the polysaccharide (29-32). As Figure 3b shows all peaks of zeolite have appeared in the hydrogel composite with no significant shift or new peaks, which indicates the success of the full incorporation of zeolite into the hydrogel structure. Swelling kinetics is a critical factor for wound healing materials providing a wet environment for wound healing. Moreover, water-absorbing structures absorb wound exudates and improve the healing process. The swelling rates of the alginate and hydrogel nanocomposite were 247 and 130%, respectively, which reached their maximum during 6 hr (Figure 4a). 

The biodegradability of the hydrogels was evaluated in PBS (pH; 7.4) and the results are depicted in Figure 4b. The results showed that around 40% of the initial weight of the pure Alg and Alg/Zeo hydrogel was lost during 7 days. 

***Antibacterial activity***

In order to evaluate the antibacterial activity of the fabricated hydrogels, we employed the micro broth dilution method (33). All investigated strains were completely resistant and the fabricated hydrogel did not induce antibacterial activity, unlike previous reports on the antibacterial activity of clinoptilolite zeolites.

***Hemocompatibility***

The hemocompatibility of the hydrogels was evaluated based on the hemolysis induction method. As shown in Figure 5, alginate hydrogel did not cause any significant hemolysis and showed hemocompatibility. While zeolite alone and when embedded in alginate hydrogel causes 3.95% and 4.2% hemolysis, respectively at a concentration of 20 mg/ml. This value is negligible compared with another study that reported hemolysis of 5.7% for zeolite and 2.6 % when embedded into graphene sheets at a concentration of 1 mg/ml (34). 

***Cell viability***

The viability of L929 fibroblast cells was evaluated according to the indirect MTT assay using 48 hr extracts of the samples (pure zeolite (Figure 6a) and Alg/Zeo hydrogels (Figure 6b)). The results showed that a high concentration of zeolite (100 and 50 mg/ml, both in pure and composited with Alg) induced significant toxicity (*P*<0.01). In pure form, 25 mg/ml zeolite induced significant toxicity (*P*<0.01) while the composite form (Alg/Zeo) exhibited negligible toxicity (*P*<0.1) indicating that Alg hydrogel improved the biocompatibility of zeolite. Moreover, pure and nanocomposite forms of zeolite at 12.5 and 6.25 mg/ml were biocompatible. 

***Animal study results***

The wound-healing efficacy of the fabricated hydrogels was evaluated in the burned wound-healing model in rats. The healing process was evaluated by monitoring the wound contraction for 14 days (Figure 7) and histopathological assessments (Figures 8, 9, and 10). The resultant wound closure implied that the treatment using pure Alg and Alg/Zeo hydrogel significantly (*P*<0.01) improved wound contraction compared with the control group. These findings revealed that the body is unable to cure the burn wound by itself and requires an effective treatment to facilitate the healing process. 

Quantitative data regarding histopathological assessment according to the employed criteria on days 7 and 14 are shown in Figures 8 and 10. Compared with the control group, in the Alg and Alg/Zeo groups on day 7, the epidermal and granulation tissue layers were significantly thicker (*P*<0.001 and *P*<0.05). Regarding the basal cell layer, most of the basal cells of the Alg group tended to be columnar and close together (Figure 9). 

However, no significant difference was observed between basal cell layers of the control and Alg/Zeo groups (*P*>0.05). In addition, angiogenesis was observed at a greater level compared with the control group in the dermis of the entire wound area of the Alg and Alg/Zeo groups on day 7 (*P*<0.001). Although Alg and combination of Alg and zeolite accelerated the wound healing process, there was no remarkable difference between the healing rates of the corresponding groups (*P*>0.05). On day 14, the wound re-epithelialization was almost completed (86±4%) in the Alg group with a significant difference from the control group (*P*<0.001). Moreover, there was a significant difference between the re-epithelialization of Alg/Zeo and control groups (*P*<0.001). On this day, in the Alg group, most basal cells were columnar in a single and closely arranged layer. However, in the Alg/Zeo group, most basal cells tended to be columnar and close together (Figure 9). 

As presented in Figure 8, on day 14, there were significant differences between the basal cell layer of Alg and Alg/Zeo groups with the control group (*P*<0.01 and *P*<0.05, respectively). Regarding the granulation tissue layer on the 14^th^ day, there were significant differences between the control group and other groups (*P*<0.001 for the Alg group and *P*<0.05 for the Alg/Zeo group). Compared with the control group, the angiogenesis level, on the 14^th^ day, was significantly higher in the other groups (*P*<0.001). As shown in Figure 8, there was no significant difference (*P*>0.05) in the percentage of neutrophils among all groups at any time point. Also as expected, with the passage of time from the 7^th^ day to the 14^th^, the number of neutrophils steadily decreased. In addition, the lowest neutrophil count was seen in the Alg group. On the 7^th^ day, a significant difference (*P*<0.05) was observed between Cnt with Alg and Alg+Zeo groups in the macrophage number, however, there was no significant difference between them on the 14th day (*P*>0.05). As can be seen, the macrophage count was higher in the Cnt group as compared with that in the other groups on all days (Figure 8). Interestingly, it should be noted that in terms of the studied variables, no significant difference was seen between Alg and Alg/Zeo groups on days 7 and 14 (*P*>0.05). The collagen density was evaluated using Masson’s trichrome staining on day 14. The collagenization rate was significantly different in the Cnt group from the others (*P*<0.01). Wounds treated with Alg showed greater collagen deposition and organization as compared with other wounds (Figure 10).

## Discussion

Hydrogels have been widely explored as a scaffold due to mimicking the natural extracellular matrix in drug delivery, tissue engineering, cell culture, and wound healing with antibacterial properties (35). Zeolites have been dispersed and evaluated as fillers in a polymer matrix for biomedical applications (36). In our study, zeolite as a nanostructure filler was integrated into Alg hydrogel and characterized. The hydrodynamic and crystalline size of the zeolite was obtained at 367 ± 0.2 and 41 nm, respectively. The morphology of the fabricated hydrogels showed a porous structure. Porosity is a critical property for tissue engineering scaffolds. For wound healing applications, the porous structure allows waste/nutrient exchange, as well as gaseous exchange between the wound bed and the environment (37, 38). As shown in Figure 4, the hydrogel nanocomposite had a lower swelling rate compared with alginate hydrogel. This reduction in swelling can be attributed to the blockage of Alg water-absorbing sites by the zeolite crystals. Although studies reported increasing water absorption in the presence of zeolites, a study also showed that water absorption of gelatin film containing zeolite did not increase and polymeric structure absorbed a larger amount of water (39). However, it seems that this amount of water absorption by hydrogel nanocomposites is suitable for absorbing exudate from the wound or maintaining a moist environment. The swelling kinetics depends on the density and accessibility of hydrophilic/water-absorbing sites/functional groups (40-42). Some other researchers have shown that the incorporation of nanostructures into Alg hydrogel reduced swelling kinetics (43, 44). Zhang *et al*. fabricated pH-sensitive Alg/hydroxyapatite (Alg/HA) nanocomposite beads and observed a reduction in swelling upon the incorporation of HA crystals (43). The biodegradability obtained results of hydrogel nanocomposite was around 40% during 7 days, which shows acceptable wound healing/dressing materials since the wound healing process is monitored for 14 days and refreshes at specific time points. The ion exchange processes between Na^+^ ions present in PBS solution and Alg cross-linker (Ca^+2^) gradually destabilize the cross-linking and result in the disintegration of gel and dissolution of the polymer (45). Our results showed that this type of unmodified zeolite (clinoptilolite) did not have any antibacterial activity. Researchers showed that unmodified clinoptilolite does not have antimicrobial activity, but it can be used as a matrix carrier for the Cetylpyridinium cation (46). Neethu Ninan *et al*. compared the antibacterial effect of gelatin/faujasite, mineral groups of the zeolite, with gelatin/faujasite-copper composite. The results proved that the gelatin/faujasite composite did not show any antibacterial activity (47). Clinoptilolite has various applications in antimicrobial, anticancer, and antiviral activity (48). Zeolites, due to their adsorption and ion exchange capabilities have been used for drug delivery systems, which means that the mechanism of zeolite to effect, depends on an ion-exchanging process (20, 49). For the antibacterial effect, silver ion is the most common ion used in the exchange process (20). A study reported that chitosan including zeolites/Ag^+^ showed better antibacterial activity compared with chitosan/Ag^+^. Zeolites can improve the antibacterial property of metallic ions by providing controlled release and prevention of oxidation of loaded metal ions within their structure (49, 50). Also, it is mentioned that zeolites, generally have an antibacterial effect which can be increased when used with other complementary elements or loaded by antibacterial drugs, so that when gentamicin is loaded in ZSM-5 zeolite the antibacterial effect of the gentamicin increases four times compared with gentamicin with hydroxyapatite alone (51). Gelatin/clinoptilolite-Ag is reported as a promising wound dressing with great antibacterial properties (39). The viability of L929 fibroblast cells during 48 hr of treatment with hydrogel nanocomposite containing various concentrations of zeolite showed no significant toxicity up to 25 mg/ml. It is reported that zeolites are not carcinogenic to humans, have no negative effects on the physiological systems of the body, and have no toxicity effects (22, 39). Also, a study showed that cell viability was not affected by the addition of Na-zeolite to LM pectin hydrogel, so the viability and cell proliferation were the same between LM pectin hydrogel and hydrogel with Na-zeolite (52). In scaffolds, zeolite can act as a reservoir of oxygen, to deliver oxygen to the cells. The scaffold containing fluorinated zeolite Y crystals with polyurethane was fabricated with the aim of vascular replacement. These scaffolds in comparison with zeolite-free scaffolds enhanced the proliferation activity of human coronary artery smooth muscle cells. This increase was attributed to the oxygen delivery to the cells by zeolite particles (53). In *in vivo* study, our results showed that the alginate-zeolite group did not show significant results compared with the alginate group in the wound healing treatment. This can be attributed to the reduction of the volume fraction of the alginate matrix due to the dose of zeolite, the exothermic effect, or the lack of use of modified zeolite. It can be concluded that zeolites can have an effect depending on the type, size, concentration, and type of ion-exchange, which could induce a significant beneficial effect in the composite for medical usage. These mentioned influential parameters are given in the results of other researchers. Salehi *et al*. presented a wound dressing comprising nano-zeolite (20-90 nm) and chamomile extract loaded in the starch-based hydrogel scaffolds to accelerate the healing process. The results of animal and clinical steps of their study indicated promoted granulation tissue and epithelialization of the ulcers when starch/extract hydrogel was used with 4 wt% nano-zeolite (54). In another study, composite scaffolds including gelatin/copper activated faujasites (CAF), mineral groups of the zeolite family, were fabricated to promote partial thickness wound healing. They showed that a composite containing 0.5% CAF develops deposition of collagen in the dermis and good keratinocyte infiltration in the epidermis which result in enhanced oxygen supply and antibacterial effect of CAF, therefore they noted that this composite can be an ideal candidate for wound healing applications (47). A study mentioned that severe thermal injury and necrosis of surrounding tissues could occur when the naked zeolite is used due to an exothermic reaction that raises the temperature of the wound. So, they offer a composite strategy, a graphene sponge with good thermal conductivity to eliminate its thermal injury (34).

## Conclusion

Development of nanostructured biomaterials has had unprecedented effects on the treatment of various diseases and damages. Wound healing is a multistep and complicated process and any interference or delays in each step may induce chronic wound progression. Accordingly, various attempts have been made to help and improve the wound healing process. In this scenario, wound healing/dressing materials have shown promising results and different types of structures fabricated from a wide variety of sources have been developed as the healing/dressing materials. Hydrogel-based biomaterials provide fascinating properties beneficial for the wound healing process, such as high water content (supporting the wet wound healing process), high hydrophilicity (beneficial for wound exudates absorption), porosity (suitable for nutrient/waste/gaseous exchange), and gel formulation (providing the ability to cover whole wound surface). Polysaccharides-based hydrogels, such as alginate, are well-studied structures with a huge potential for wound healing/management applications. Moreover, nanocomposite hydrogels are sophisticated biomaterials integrating the sutural properties of hydrogels with nanoscale features. The wound-healing efficacy of pristine (unmodified) clinoptilolite (a natural zeolite) has not been evaluated in an animal model. In the present work, we integrated zeolite with Alg hydrogel as the wound healing materials. The characterizations revealed that incorporation of zeolite did not cause any significant effects (antibacterial and wound healing effects). It must be noticed that zeolites have micro-and nano feature structures applicable for drug delivery and regenerative medicine. In addition, the incorporation of zeolite makes nanofeatures into bulk objects (e.g., hydrogels) and it can be beneficial for various applications such as regenerative medicine. For future studies, various surface modifications and doping in clinoptilolite can be conducted to develop more bioactive zeolite to meet the observed shortcomings of the present study. 

## Authors’ Contributions

HS helped with investigation, data curation, formal analysis, and writing the original draft. R F helped investigate, conceptualize, write, review, edit, and with formal analysis. M S, ML, HHN, SMK, and NRK performed investigation, data curation, and experiments. AS, SZ, and MAS helped with formal analysis, writing, review, and editing. SMA performed formal analysis, and helped write, review, and edit. MJMP performed conceptualization, methodology, visualization, supervision, formal analysis, helped write the original draft, and with writing, review, and editing. 

## Etical Statement

This study has been approved by the Ethics Committee of Kerman University of Medical Sciences, Kerman, Iran (IR.KMU.REC.1400.479). 

## Conflicts of Interest

The author(s) declared no potential conflicts of interest with respect to the research, authorship, and/or publication of this article.

## References

[1] Shah SA, Sohail M, Khan S, Minhas MU, De Matas M, Sikstone V (2019). Biopolymer-based biomaterials for accelerated diabetic wound healing: a critical review. Int J Biol Macromol.

[2] Davison-Kotler E, Marshall WS, García-Gareta E (2019). Sources of collagen for biomaterials in skin wound healing. Bioengineering.

[3] Bianchera A, Catanzano O, Boateng J, Elviri L (2020). The place of biomaterials in wound healing. Ther Dress Wound Heal Appl.

[4] Pourshahrestani S, Zeimaran E, Kadri NA, Mutlu N, Boccaccini AR (2020). Polymeric hydrogel systems as emerging biomaterial platforms to enable hemostasis and wound healing. Adv Healthc Mater.

[5] Ding C, Tian M, Feng R, Dang Y, Zhang M (2020). Novel self-healing hydrogel with injectable, pH-responsive, strain-sensitive, promoting wound-healing, and hemostatic properties based on collagen and chitosan. ACS Biomater Sci Eng.

[6] Salehi M, Zamiri S, Samadian H, Ai J, Foroutani L, Ai A (2021). Chitosan hydrogel loaded with aloe vera gel and tetrasodium ethylenediaminetetraacetic acid (EDTA) as the wound healing material: in vitro and in vivo study. J Appl Polym Sci.

[7] Samadian H, Khastar H, Ehterami A, Salehi M (2021). Bioengineered 3D nanocomposite based on gold nanoparticles and gelatin nanofibers for bone regeneration: in vitro and in vivo study. Sci Rep.

[8] Murray RZ, West ZE, Cowin AJ, Farrugia BL (2019). Development and use of biomaterials as wound healing therapies. Burns Trauma.

[9] Nazarnezhada S, Abbaszadeh-Goudarzi G, Samadian H, Khaksari M, Ghatar JM, Khastar H (2020). Alginate hydrogel containing hydrogen sulfide as the functional wound dressing material: in vitro and in vivo study. Int J Biol Macromol.

[10] Mohammadpour M, Samadian H, Moradi N, Izadi Z, Eftekhari M, Hamidi M (2022). Fabrication and characterization of nanocomposite hydrogel based on alginate/nano-hydroxyapatite loaded with linum usitatissimum extract as a bone tissue engineering scaffold. Mar Drugs.

[11] Kurakula M, Rao GK, Kiran V, Hasnain MS, Nayak AK (2020). Alginate-based hydrogel systems for drug releasing in wound healing. Alginates Drug Deliv.

[12] Liao J, Jia Y, Wang B, Shi K, Qian Z (2018). Injectable hybrid poly (ε-caprolactone)-b-poly (ethylene glycol)-b-poly (ε-caprolactone) porous microspheres/alginate hydrogel cross-linked by calcium gluconate crystals deposited in the pores of microspheres improved skin wound healing. ACS Biomater Sci Eng.

[13] Thomas A, Harding K, Moore K (2000). Alginates from wound dressings activate human macrophages to secrete tumour necrosis factor-α. Biomaterials.

[14] Yang D, Jones KS (2009). Effect of alginate on innate immune activation of macrophages. J Biomed Mater Res A.

[15] Ninan N, Muthiah M, Park I-K, Wong TW, Thomas S, Grohens Y (2015). Natural polymer/inorganic material based hybrid scaffolds for skin wound healing. Polym Rev.

[16] Iqbal N, Kadir MRA, Mahmood NHB, Yusoff MFM, Siddique JA, Salim N (2014). Microwave synthesis, characterization, bioactivity and in vitro biocompatibility of zeolite–hydroxyapatite (Zeo–HA) composite for bone tissue engineering applications. Ceram Int.

[17] McDonnell AM, Beving D, Wang A, Chen W, Yan Y (2005). Hydrophilic and antimicrobial zeolite coatings for gravity-independent water separation. Adv Func Mater.

[18] Tondar M, Parsa MJ, Yousefpour Y, Sharifi AM, Shetab-Boushehri SV (2014). Feasibility of clinoptilolite application as a microporous carrier for pH-controlled oral delivery of aspirin. Acta Chimica Slovenica.

[19] Hovhannisyan VA, Dong C-Y, Lai F-J, Chang N-S, Chen S-J (2018). Natural zeolite for adsorbing and release of functional materials. J Biomed Opt.

[20] Demirci S, Ustaoğlu Z, Yılmazer GA, Sahin F, Baç N (2014). Antimicrobial properties of zeolite-X and zeolite-A ion-exchanged with silver, copper, and zinc against a broad range of microorganisms. Appl Biochem Biotechnol.

[21] Concepción-Rosabal B, Rodríguez-Fuentes G, Bogdanchikova N, Bosch P, Avalos M, Lara V (2005). Comparative study of natural and synthetic clinoptilolites containing silver in different states. Microporous Mesoporous Mater.

[22] Cerri G, Farina M, Brundu A, Daković A, Giunchedi P, Gavini E (2016). Natural zeolites for pharmaceutical formulations: Preparation and evaluation of a clinoptilolite-based material. Microporous Mesoporous Mater.

[23] Koyama K, Takeuchi Y (1977). Clinoptilolite: the distribution of potassium atoms and its role in thermal stability. Zeitschrift für Kristallographie-Crystalline Materials.

[24] Zhang M, Sun L, Wang X, Chen S, Kong Y, Liu N (2014). Activin B promotes BMSC-mediated cutaneous wound healing by regulating cell migration via the JNK—ERK signaling pathway. Cell Transplant.

[25] Baghbanian SM, Rezaei N, Tashakkorian H (2013). Nanozeolite clinoptilolite as a highly efficient heterogeneous catalyst for the synthesis of various 2-amino-4 H-chromene derivatives in aqueous media. Green Chemist.

[26] Treacy MM, Higgins JB (2007). Collection of simulated XRD powder patterns for zeolites fifth (5th) revised edition.

[27] Olad A, Doustdar F, Gharekhani H (2018). Starch-based semi-IPN hydrogel nanocomposite integrated with clinoptilolite: preparation and swelling kinetic study. Carbohydr polym.

[28] Dinu MV, Lazar MM, Dragan ES (2017). Dual ionic cross-linked alginate/clinoptilolite composite microbeads with improved stability and enhanced sorption properties for methylene blue. React Funct Polym.

[29] Rashidzadeh A, Olad A, Salari D (2015). The effective removal of methylene blue dye from aqueous solutions by NaAlg-g-poly (acrylic acid-co-acryl amide)/clinoptilolite hydrogel nanocomposite. Fibers Polym.

[30] Khalid I, Ahmad M, Minhas MU, Barkat K (2018). Preparation and characterization of alginate-PVA-based semi-IPN: Controlled release pH-responsive composites. Polym Bull.

[31] Iqbal B, Muhammad N, Jamal A, Ahmad P, Khan ZUH, Rahim A (2017). An application of ionic liquid for preparation of homogeneous collagen and alginate hydrogels for skin dressing. J Mol Liq.

[32] Manjula B, Varaprasad K, Sadiku R, Raju KM (2013). Preparation and characterization of sodium alginate–based hydrogels and their in vitro release studies. Adv Polym Technol.

[33] Kobayashi I, Muraoka H, Saika T, Nishida M, Fujioka T, Nasu M (2004). Micro-broth dilution method with air-dried microplate for determining MICs of clarithromycin and amoxycillin for Helicobacter pylori isolates. J Med Microbiol.

[34] Liang Y, Xu C, Liu F, Du S, Li G, Wang X (2019). Eliminating heat injury of zeolite in hemostasis via thermal conductivity of graphene sponge. ACS Appl Mater Interfaces.

[35] Sang Y, Li W, Liu H, Zhang L, Wang H, Liu Z (2019). Construction of nanozyme-hydrogel for enhanced capture and elimination of bacteria. Adv Funct Mater.

[36] Taaca KLM, Olegario EM, Vasquez MR (2019). Impregnation of silver in zeolite–chitosan composite: thermal stability and sterility study. Clay Miner.

[37] Tavakolian M, Munguia-Lopez JG, Valiei A, Islam MS, Kinsella JM (2020). Highly absorbent antibacterial and biofilm-disrupting hydrogels from cellulose for wound dressing applications. ACS Appl Mater Interfaces.

[38] Fan Z, Liu B, Wang J, Zhang S, Lin Q, Gong P (2014). A novel wound dressing based on Ag/graphene polymer hydrogel: effectively kill bacteria and accelerate wound healing. Adv Funct Mater.

[39] Hubner P, Donati N, de Menezes Quines LK, Tessaro IC, Marcilio NR (2020). Gelatin-based films containing clinoptilolite-Ag for application as wound dressing. Mater Sci Eng C.

[40] Karoyo AH, Wilson LD (2021). A review on the design and hydration properties of natural polymer-based hydrogels. Materials.

[41] Ahmed EM (2015). Hydrogel: preparation, characterization, and applications: a review. J Adv Res.

[42] Mahon R, Balogun Y, Oluyemi G, Njuguna J (2020). Swelling performance of sodium polyacrylate and poly (acrylamide-co-acrylic acid) potassium salt. SN Appl Sci.

[43] Zhang J, Wang Q, Wang A (2010). In situ generation of sodium alginate/hydroxyapatite nanocomposite beads as drug-controlled release matrices. Acta Biomater.

[44] Fan L, Zhang J, Wang A (2013). In situ generation of sodium alginate/hydroxyapatite/halloysite nanotubes nanocomposite hydrogel beads as drug-controlled release matrices. J Mater Chem B.

[45] Bajpai S, Sharma S (2004). Investigation of swelling/degradation behaviour of alginate beads crosslinked with Ca2+ and Ba2+ ions. React Funct Polym.

[46] Milyovich S, Pantyo V, Danko E, Pogodin A, Filep M, Fizer O (2023). Antibacterial application of carpathian clinoptilolite as cetylpyridinium carrier. Biointerface Res Appl Chem.

[47] Ninan N, Muthiah M, Yahaya NAB, Park I-K, Elain A, Wong TW (2014). Antibacterial and wound healing analysis of gelatin/zeolite scaffolds. Colloids Surf B.

[48] de Gennaro B, Catalanotti L, Cappelletti P, Langella A, Mercurio M, Serri C (2015). Surface modified natural zeolite as a carrier for sustained diclofenac release: a preliminary feasibility study. Colloids Surf B.

[49] Taaca KLM, Vasquez Jr MR (2017). Fabrication of ag-exchanged zeolite/chitosan composites and effects of plasma treatment. Microporous Mesoporous Mater.

[50] Montallana ADS, Cruz CEV, Vasquez Jr MR (2018). Antibacterial activity of copper-loaded plasma-treated natural zeolites. Plasma Med.

[51] Serati-Nouri H, Jafari A, Roshangar L, Dadashpour M, Pilehvar-Soltanahmadi Y, Zarghami N (2020). Biomedical applications of zeolite-based materials: a review. Mater Sci Eng C.

[52] Kocaaga B, Kurkcuoglu O, Tatlier M, Batirel S, Guner FS (2019). Low-methoxyl pectin–zeolite hydrogels controlling drug release promote in vitro wound healing. J Appl Polym Sci.

[53] Bacakova L, Vandrovcova M, Kopova I, Jirka I (2018). Applications of zeolites in biotechnology and medicine–a review. Biomater Sci.

[54] Salehi H, Mehrasa M, Nasri-Nasrabadi B, Doostmohammadi M, Seyedebrahimi R, Davari N (2017). Effects of nanozeolite/starch thermoplastic hydrogels on wound healing. J Res Med Sci.

